# Laparoscopic and robot-assisted suture versus mesh hysteropexy: a retrospective comparison

**DOI:** 10.1007/s00192-022-05283-6

**Published:** 2022-07-26

**Authors:** Deepa Gopinath, Chin Yong, Sam Harding-Forrester, Felix McIntyre, Dean McKenzie, Marcus Carey

**Affiliations:** 1 Nepean Clinical School, Nepean Hospital, Sydney, Kingswood 2747 Australia; 2 Epworth HealthCare, Melbourne, Australia; 3 University of Melbourne, Melbourne, Australia

**Keywords:** Uterine prolapse, Uterine preservation, Uterine suspension, Uterosacral plication

## Abstract

**Introduction and hypothesis:**

Our study was aimed at comparing the outcomes of laparoscopic and robot-assisted laparoscopic suture-based hysteropexy (SutureH) versus sacral hysteropexy using mesh (MeshH) for bothersome uterine prolapse. Our hypothesis is that MeshH is more successful and provides better uterine support than SutureH.

**Methods:**

A retrospective cohort study of 228 consecutive women who underwent re-suspension of the uterus using uterosacral ligaments (SutureH *n*=97) or a “U-shaped” mesh from the sacral promontory (MeshH, *n*=132). Surgery was performed by laparoscopy or robot-assisted laparoscopy. Subjects were assessed at baseline, 1 year, and beyond 1 year. The null hypothesis, that SutureH and MeshH have similar success, was based on a composite outcome (“composite success”), and that they provide the same level of uterine support, was based on POP-Q point C at 1 year. “Composite success” was defined as: POP-Q point C above the hymen; absence of a vaginal bulge; no repeat uterine prolapse surgery or pessary placement. Other outcomes included improvement in symptomology using Patient Global Impression of Improvement, POP-Q point C change and complications.

**Results:**

Follow-up data were available for 191 out of 228 women. “Composite success” was not significantly different between MeshH and SutureH groups (81.7% vs 84.5%, *p*=0.616). MeshH provided better elevation of the uterus than SutureH (point C change: −7.38cm vs −6.99cm; *p*<0.001). Similar symptom improvement and low complications occurred in both groups.

**Conclusions:**

Laparoscopic and robot-assisted laparoscopic suture hysteropexy and mesh sacral hysteropexy provide women with minimally invasive, durable surgical options for uterine preservation. “Composite success” was similar in the two groups, but MeshH provided better uterine support than SutureH. However, SutureH gives women an effective mesh-free option.

## Introduction

Pelvic Organ Prolapse (POP) is a common condition with a 19% lifetime risk of surgery in Australian women [1]. In epidemiological studies, the prevalence of apical repair during surgery for POP varies from of 14.2% to 70% [2, 3]. The uterus and upper vagina receive direct support from the uterosacral and cardinal ligaments. The dorsal attachment of uterosacral ligaments to the parietal fascia of the piriformis muscle prevents the uterus and upper vagina from prolapsing downwards through the levator hiatus [4]. Although the collagenous content of this ligament is variable, it has proven tensile strength when used for vaginal vault fixation [5, 6]. Traditionally, uterine prolapse has been treated by vaginal hysterectomy and McCall culdoplasty. A large Australian population-based cohort study found that the risk of re-operation for prolapse increases every decade after vaginal hysterectomy, with a risk of 12.2% at 30 years [7].

Given the passive role that the uterus plays in apical prolapse, re-suspension of the uterus (hysteropexy) instead of hysterectomy should be adequate. Although historically offered as a fertility-sparing procedure, increasingly more women prefer uterine-preserving surgeries [8]. A recent systematic review comparing hysteropexy with hysterectomy found that hysteropexy had similar short-term outcomes with less blood loss, operating, and mesh exposure than hysterectomy and should be offered as an option [9].

Many techniques including vaginal, abdominal and laparoscopic approaches exist for hysteropexy [10]. Performing hysterectomy and any associated vaginal repair via a vaginal approach is convenient and time efficient. Laparoscopy has the advantage of a superior visualisation of the ureters and hypogastric plexus, but a longer operating time. Abdominal hysteropexy has no advantage over the vaginal and laparoscopic approaches [9]. Currently, there is no standardised technique for either suture-based hysteropexy or sacral hysteropexy using mesh. Bilateral and unilateral suture-based hysteropexy have been described with objective success rates of 79% and 94% at 12 and 24 months respectively [11, 12].

Commonly described techniques for mesh sacral hysteropexy typically use polypropylene mesh attached to posterior cervix or wrapped around the cervix and fixed to the sacral promontory [10, 13]. Laparoscopic mesh sacral hysteropexy has similar subjective outcomes but better anatomical elevation and total vaginal length at 7 years compared to vaginal hysterectomy in an RCT [14]. A recent systematic review showed pooled objective success rates of 70.5% for suture-based hysteropexy and 92% for mesh-based hysteropexy, but, to our knowledge, there is no study directly comparing these two techniques [15]. Ongoing concerns about gynaecological mesh use have resulted in a renewed interest in mesh-free options for pelvic organ prolapse surgery such as suture-based hysteropexy.

The aim of our study is to compare the outcomes of laparoscopic and robot-assisted suture-based hysteropexy (SutureH) versus sacral hysteropexy using mesh (MeshH) for bothersome uterine prolapse. Our hypothesis is that MeshH is more successful and provides better uterine support than SutureH.

## Materials and methods

### Study design

This is a retrospective cohort study conducted to determine the superiority of MeshH and SutureH for “composite success” beyond 1 year and uterine support at 1 year. Surgery was performed by a single surgeon (MC) at two hospital sites. The initial follow-up was at 5 weeks and 1 year. Objective assessment was undertaken at baseline and at 1 year using the POP-Q system. Women were asked whether they experienced a sensation of a vaginal bulge at the follow-up visits. Beyond 1 year, changes in symptom severity were measured using the Patient Global Impression of Improvement (PGI-I) scale by two independent investigators who were blinded to the surgical procedure. Telephone reviews were conducted using a “cold-calling” technique without prior contact or notification to reduce participation bias.

### Participants

All women who had laparoscopic or robot-assisted laparoscopic MeshH or SutureH from September 2010 until October 2018 were included in this study. Both procedures were offered to women with bothersome uterine prolapse requesting uterus-sparing prolapse surgery and who had no contraindications for uterus preservation. Additional anterior and posterior vaginal repairs and anti-incontinence procedures were performed as indicated. Laparoscopy or robot-assisted laparoscopy surgery was dependent upon patient and surgeon preference, and on the hospital where surgery was performed, as only one of the hospitals had access to robot-assisted surgery. No sample size analysis was performed.

### Procedures

All procedures were done under general anaesthesia. Women received peri-operative antibiotics and thromboprophylaxis. Robot-assisted laparoscopic hysteropexy was done using da Vinci Xi and X systems (Intuitive Surgical, USA). In all women, a uterine manipulator and an indwelling catheter were placed at the commencement of surgery.

**Fig. 1:**
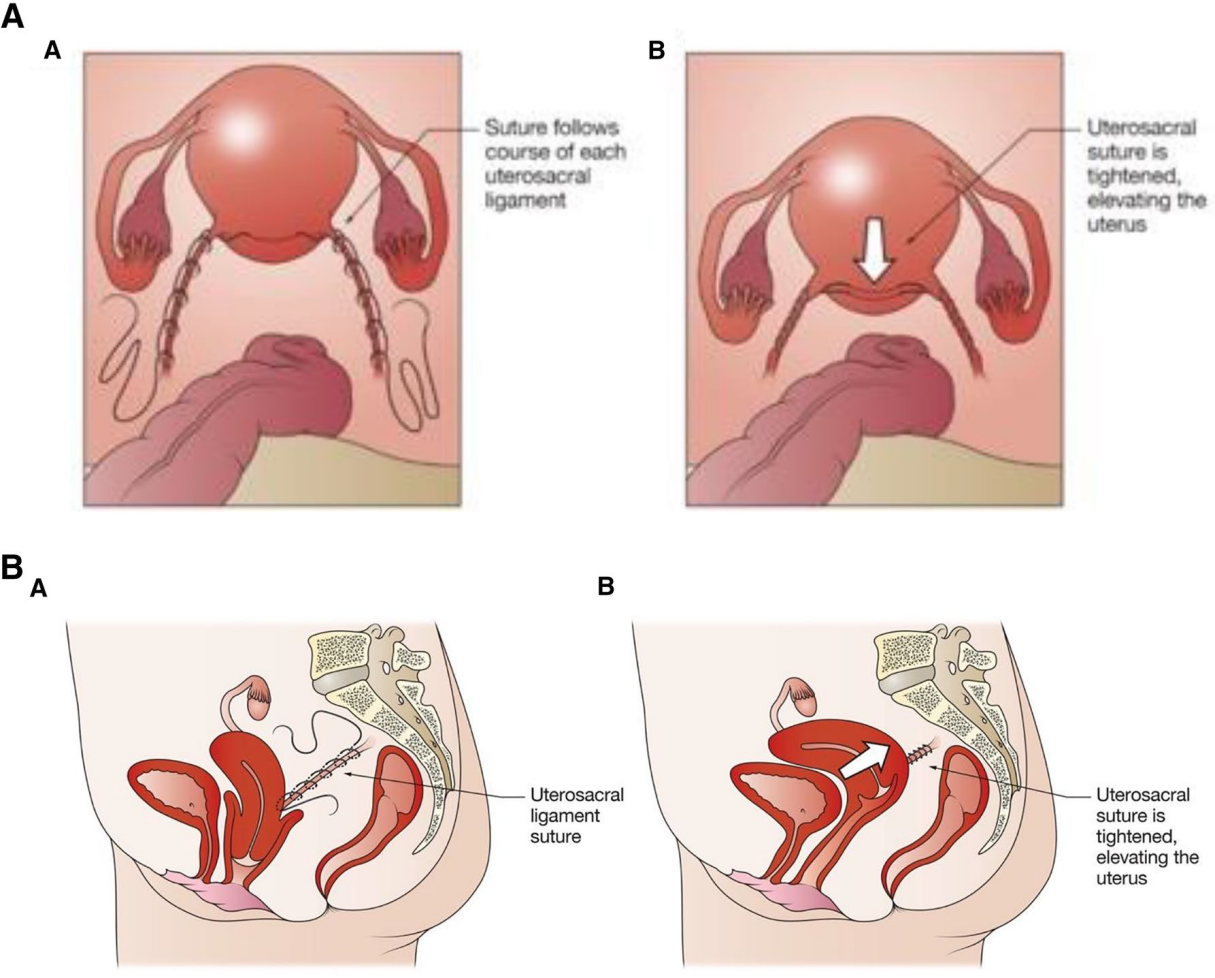
**a** During suture hysteropexy, sutures are placed into the posterior cervix and along each uterosacral ligament dorsally towards the sacrum (**A**) and, when tied (**B**), shorten the ligaments and re-support the uterus and upper vagina. **b** Lateral view of suture hysteropexy demonstrating uterosacral sutures before the sutures are tensioned and tied (**A**) and after being tied (**B**)

After laparoscopic entry and port placement, the ureters were identified on the pelvic side walls. In SutureH, the peritoneum overlying the uterosacral ligament was opened up to the level of the sacrum. A bidirectional barbed absorbable suture (Bidirectional Stratafix; Ethicon, USA) was anchored to the posterosuperior aspect of the cervix and passed twice (back and forth), in a helical fashion along the length of the ligament, shortening and plicating the ligament dorsally towards the sacrum and then towards the cervix and repeated on the other side. A second permanent 0 Prolene suture (Ethicon, USA) was passed twice (back and forth) along the shortened ligament on either side. This effectively resulted in four suture strands in each uterosacral ligament (Fig. 1).

**Fig. 2:**
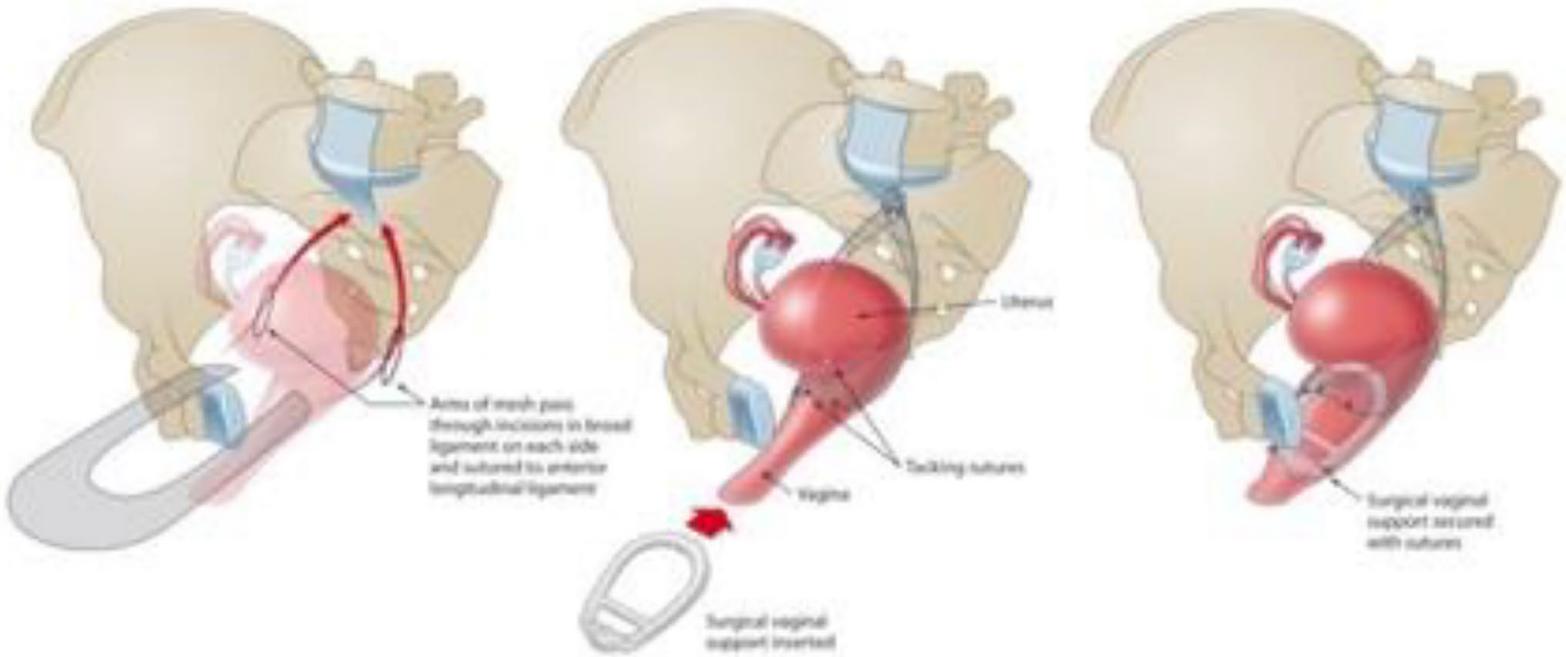
Surgical technique for mesh hysteropexy. A “U-shaped” mesh, passes through the broad ligament windows, suspends the uterus and upper vagina from the sacral promontory and a supporting pessary is placed in the vagina

In MeshH, the peritoneum over the sacral promontory and the right pelvic side wall is opened to the level of the pouch of Douglas. The uterovesical fold of the peritoneum is then dissected to expose the anterior cervix. A hand-cut U-shaped polypropylene mesh is secured with delayed absorbable sutures (2-0 PDS) on the anterior cervix. All meshes used were approved for clinical use by the Therapeutic Goods Administration (TGA), at the time that they were used in the study. Avascular windows were created through each broad ligament through which the mesh straps were passed and then anchored to the anterior longitudinal ligament on the sacral promontory with sutures (Fig. 2). The peritoneum is then closed over the mesh using a delayed absorbable suture. Additional anterior and/or posterior repairs and/or anti-incontinence procedures were undertaken as indicated.

At the completion of surgery, all patients were fitted with a post-operative surgical support pessary (S-POP, Gynaecologic Pty, Australia) which was scheduled to be removed at 4 weeks in the outpatient clinic. Vaginal packing was inserted and removed the next post-operative day.

The null hypothesis, that MeshH and SutureH have identical success, was based on a composite outcome (“composite success”) beyond 1 year, and that they provide the same level of support, was based on the position of the cervix (POP-Q point C) at 1 year. “Composite success” was defined as: POP-Q point C above 0 cm, absence of vaginal bulge symptoms and no repeat uterine prolapse surgery or pessary placement. We also assessed women’s subjective improvement in prolapse symptoms using PGI-I; POP-Q point C change; change in POP-Q scores; and complications.

Subjects’ characteristics were summarised using means and standard deviations for continuous variables, or medians and 25th and 75th percentiles where appropriate. Categorical data were presented as percentages. Binary variables, such as smoking, were analysed using the Chi-squared test, or Fisher’s exact test for small frequencies. Binary outcome variables, such as vaginal bulge symptoms, were assessed by relative risks (RR) and 95% confidence intervals (CI). Change in POP-Q scores and PGI-I were assessed using linear regression or median quantile regression, which directly compares medians [16], if indicated by the data. Both types of regression employed robust standard errors [17, 18], allowing for testing across time, and were conducted with and without adjusting for age at operation. As PGI-I was done at various time points after the 1-year follow-up, linear regression was used to adjust for time since the operation as well as age at operation. All statistical analyses were conducted by a biostatistician (DMcK) who was not involved with the original study design, employing Stata version 17 (Stata, College Station, TX, USA).

This study was a service evaluation project and did not require human ethics committee (HREC) approval.

## Results

**Fig. 3:**
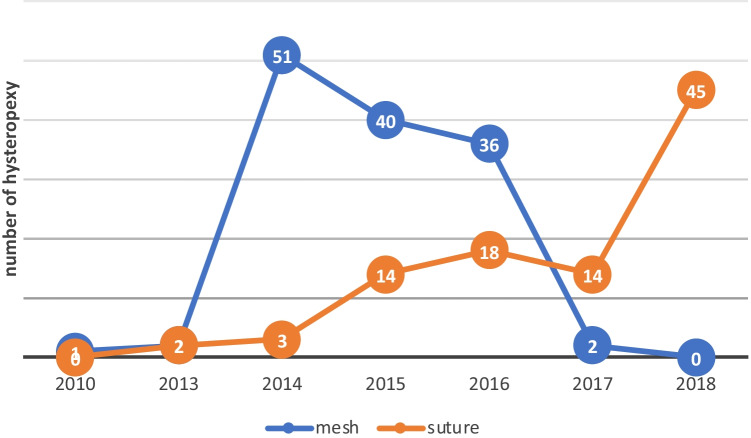
Trend in hysteropexy with time (number of cases within *circles*)

A total of 228 women underwent laparoscopic or robotic hysteropexy between September 2010 and October 2018. One hundred and thirty-two women had MeshH, and 97 had SutureH. Subjects’ characteristics at baseline are detailed in Table 1. One-year follow-up data were available for 191 women (MeshH *n*=120 (91%); SutureH *n*=71 (73%). Beyond 1 year, “composite success” was no different between the MeshH and SutureH groups (81.7%, *n*=98 vs 84.5%, *n*=60; RR = 0.97, 95% CI = 0.85–1.19, *p*=0.616). When breaking down the composite success into individual domains, there was no difference between MeshH and SutureH with regard to point C ≥1 99.2% (*p*=119) vs 95.8% (*n*=68; *p*=0.113; RR 0.20 (95% CI 0.002–1.86); repeat surgery for point C 96.7% (*n*=116) vs 94.4% (*n*=67; *p*= 0.443; RR 0.59 (95% CI 0.15–2.29), placement of pessary 99.2% (*n*=119) vs 97.2% (*n*=69; *p*=0.29; RR 0.30 (95% CI 0.03–3.20) and symptoms of bulge/prolapse 81.7%(*n*=98) vs 84.5% (*n*=60; *p*=25; RR 1.18 (95% CI 0.61–2.29). At 1 year compared with baseline, MeshH provided better uterine support than SutureH (POP-Q point C: −7.38cm vs −6.99; *p*<0.001, age-adjusted, although adjusting for age made very little difference to the results. The trend of the procedures with time is shown in Fig. 3.

**Table 1:** Baseline characteristics of the two groups

Parameter	MeshH (*n*=120)	SutureH (*n*=71)	*p* value
Age in years, mean (SD) minimum, maximum, range	54.05 (16) (33–71)	48.5 (10.6) (24–72)	**<0.001**
Type of surgery, *n* (%)			
Robotic	50 (41.7)	14(19.7)	**0.002**
Laparoscopic	70 (58.3)	57 (80.3)	
Concomitant procedures, *n* (%)			
Vaginal repair	114 (95)	71 (100)	0.86
Incontinence	28 (23.3)	33 (46.5)	**<0.001**
Burch	3 (9)	17 (51.5)	**<0.001**
MUS	25(91)	15 (45.5)	0.961
Bulkamid		1 (3)	
Removal of MUS		1	
Division of MUS		1	
Smoking status, *n* (%)			
Smoker	7 (5.9)	3.(4.2)	0.746*
Non smoker	112 (94.1)	68(95.8)	
Unknown	1		
Parity, mean (SD), range	2.5 (1.3), 0–9	2.4 (1.1), 0–7	0.655
Vaginal births, mean (SD), range	2.4 (1.3), 0–9	2.2 (1.2), 0–7	0.461
Menopausal status, *n* (%)			
Post-menopausal	70 (58.3)	25 (35.2)	**0.002**
Pre-menopausal	50 (41.7)	46 (64.8)	
Previous surgery *n* (%)			
Prolapse	17 (11.7)	6 (4.2)	0.275
Incontinence	3 (2.5)	1 (1.4)	
Both	3 (2.5)	3 (4.2)	
Neither	100 (83.3)	64 (90.1)	
Baseline POPQ, mean (SD)			
Aa	1.11 (1.41)	0.34 (1.16)	**<0.001**
Ba	1.48 (1.67)	0.42 (1.25)	**<0.001**
C	−0.19 (2.49)	−2.04 (1.85)	**<0.001**
D	−1.66 (1.51)	−2.65 (1.00)	**<0.001**
Ap	1.00 (0.2)	1.00 (0.1)	>0.999**
Bp	1.00 (0.2)	1.00 (0.1)	>0.999**
GH	3.78 (0.92)	3.77 (0.76)	0.903
PB	3.60 (0.69)	3.74 (0.50)	0.14
TVL	8.86(0.94)	8.36 (0.84)	**<0.001**

Statistically significant values are in bold

*Fisher’s exact test, otherwise Chi-squared test

**Median quantile regression, median and 25th and 75th percentiles, otherwise independent-samples *t* test

Women in the MeshH group had a higher average age and were more likely to undergo robot-assisted surgery than the SutureH group. Most women in both groups had additional vaginal repairs but concomitant anti-incontinence surgery was more prevalent during SutureH. One woman had complete removal of a trans-obturator tape (TOT) along with SutureH, and another had division of tension-free vaginal tape (TVT). Meshes used for MeshH included Physiomesh (Ethicon, USA), Ultrapro (Ethicon, USA), Restorelle (Coloplast, Denmark) and TiMESH (PFM, Germany). Baseline POP-Q findings demonstrated that the MeshH group had more advanced prolapse in all compartments than SutureH. Total vaginal length, genital hiatus and perineal body measurements were similar in the two groups.

Table 2 shows the mean change in POP-Q scores. MeshH provided a greater change in POP-Q point C at 1 year compared with baseline than SutureH, even when adjusting for age at the time of surgery (*p* < 0.001). MeshH provided better anterior compartment and posterior fornix support than SutureH. There was no difference in the posterior compartment, excluding the posterior fornix, despite the SutureH group having a higher number of concomitant Burch colposuspension procedures. Vaginal length was preserved in both groups.

**Table 2:** Mean change in POP-Q scores (1 year minus baseline). Linear regressions with and without adjustment for age

POP-Q points	MeshH (*n*=120)			SutureH (*n*=71)			Difference in change, 95% CI	*p* value, unadjusted	*p* value, adjusting for age
	Baseline	1 year	Change	Baseline	1 year	Change			
Aa	1.11	−1.81	−2.92	0.34	−2.06	−2.39	−0.53 −0.94 to −0.11	**0.013**	**0.013**
Ba	1.48	−1.80	−3.29	0.42	−1.94	−2.37	−0.92 −1.38 to −0.46	**<0.001**	**<0.001**
C	−0.19	−7.38	−7.20	−2.04	−6.99	−4.92	−2.24 −3.01 to −1.47	**<0.001**	**<0.001**
D	−1.66	−7.63	−5.97	−2.65	−7.63	−4.99	−0.99 −1.56 to −0.41	**0.001**	**0.001**
Ap	0.95	−2.48	−3.43	0.65	−2.58	−3.23	−0.20 −0.65 to 0.24	0.370	0.370
Bp	0.97	−2.45	−3.45	0.65	−2.73	−3.38	−0.07 −0.41 to −0.28	0.707	0.708
TVL	8.86	8.46	−0.40	8.36	8.28	−0.08	−0.32 −0.72 to 0.08	0.116	0.116
GH	3.78	3.36	−0.42	3.77	3.16	−0.61	−0.18 −0.43 to 0.16	0.159	0.159
PB	3.60	3.81	0.21	3.74	3.98	0.24	−0.03 −0.24 to 0.18	0.775	0.774

Statistically significant values are in bold

Repeat surgery for recurrent uterine prolapse was similar in the two groups. The time from initial surgery to repeat surgery for recurrent uterine prolapse ranged from 7 to 24 months. In the MeshH group, three laparoscopic mesh-shortening procedures and a repeat mesh hysteropexy were performed for the four cases of recurrent uterine prolapse. In the SutureH group, 3 women chose a repeat SutureH and 1 had robot-assisted MeshH. At 12 months, the POP-Q point C was at −4 cm in 2 subjects, −5 cm in 1 subject and 0 cm in 1 subject in the MeshH group. The POP-Q point C was at +1 cm in 2 subjects, −1 cm in 1 subject and −4 in 1 subject in the SutureH group. Subsequent anti-incontinence surgery was done for 3 women in the MeshH and 2 in the SutureH groups. Further vaginal repairs were done for 7 women in the MeshH and 4 in the SutureH groups.

Data from the PGI-I were available for 48 out of 71 (67.6%) and 78 out of 120 (65%) in the SutureH and MeshH groups respectively (Table 3). The average period, in years, from surgery to PGI-I in MeshH was 4.14 years. Mean PGI was 1.84 (SD = 1.02) for MeshH and 2.36 (SD = 1.62) for SutureH. In the MeshH group, 73 out of 78 (93.59%) reported improvement of symptoms, 4 out of 78 (5.13%) reported no change and 1 out of 78 (1.2%) reported worsening of symptoms related to prolapse on PGI-I. Similarly, 40 out of 48 (83.3%) reported improvement, 2 out of 48 (4.12%) reported no change and 6 out of 48 (12.5%) reported worsening of symptoms in the SutureH group. Although there was a slight difference in PGI without adjustment for age and time since operation (*p* = 0.047) there was no statistically significant difference in PGI-I between the groups when controlling for these variables (1.78 vs 2.36; *p* = 0.059).

**Table 3:** Patient Global Impression of Improvement (PGI-I) for MeshH versus SutureH

Mean (SD)	MeshH (*n*=78)	SutureH (*n*=48)	*p* value (linear regression)
PGI, mean (SD)	1.84 (1.02)	2.36 (1.62)	**0.047**
PGI adjusted for years from operation to PGI and age at operation, mean (95% CI)	1.79 (1.45–2.13)	2.36 (1.94–2.77)	*0.059*

Immediate complications included two cases of posterior vaginal wall haematoma in the MeshH group from posterior repair procedures, both were self-limiting without requiring blood transfusion or haematoma evacuation. One woman had a diagnostic laparoscopy at 4 weeks for pelvic pain and was noted to have significant adhesions and a hydrosalpinx. This woman had Physiomesh implanted, which has since been recalled by the TGA. One had faecal loading at 4 weeks, which resolved with aperients. All 5 women who had voiding dysfunction had anti-incontinence surgery. This complication resolved spontaneously except for one woman in the MeshH group who required MUS division at 4 months. A single mesh exposure from a MUS occurred in the SutureH with no mesh exposures in the MeshH. Other clinical problems reported after surgery included stress incontinence (MeshH 5.8%; SutureH 5.6%), overactive bladder (MeshH 4.2%; SutureH 4.2%), dyspareunia (MeshH 5.8%; SutureH 4.2%), pelvic pain (MeshH 3.3%; SutureH 5.6%), defecatory difficulty (MeshH 0.8%; SutureH 2.8%) and dysfunctional uterine bleeding (MeshH 1.7%; SutureH 1.4%). There were 2 cases of vaginal granuloma and 1 of osteitis pubis in the MeshH group.

Two women have had term deliveries after surgery. A woman after MeshH had a Caesarean section with no recurrence of symptoms. A woman after SutureH, with very much improved symptoms at 1 year (PGI-I=1) experienced recurrence of symptoms 3 months into the pregnancy. She underwent vaginal delivery at term with a view to further prolapse surgery

## Discussion

Our study demonstrates, when performed by laparoscopy and robot-assisted laparoscopy, MeshH and SutureH are safe and effective for women with bothersome uterine prolapse choosing uterus-conserving surgery. With respect to “composite success” (POP-Q point C above the hymen; absence of vaginal bulge symptoms; and no repeat apical prolapse surgery or placement of a pessary), we found no difference between MeshH and SutureH. However, this does not indicate equivalence or non-inferiority as our study was not powered to assess this outcome. MeshH provided better uterine support than suture at 1 year. When results are broken down, the “composite success” was anchored to the least successful domain being the “absence of bulge” symptoms in both groups in our study. This somewhat undermines the value of using a composite outcome. Even though MeshH provided better uterine support, the mean difference was only 0.39 cm between the two groups, which seems unlikely to be clinically important. The change in POP-Q point C at 1 year for MeshH was 2.32 cm greater than SutureH, which may reflect the higher grade of uterine prolapse at baseline in the MeshH group. Subjective satisfaction with surgery and improved symptoms were similar in the two groups. To our knowledge, this study is the first to directly compare mesh with non-mesh hysteropexy and provides further information when deciding on surgery for uterine prolapse.

Baseline data show an increasing preference towards SutureH over MeshH with time. This trend is consistent with a general reduction in mesh usage for both prolapse and anti-incontinence surgery [16, 17]. Unsurprisingly, women in the SutureH group requiring concomitant anti-incontinence surgery tended to prefer a Burch colposuspension over a MUS.

The SutureH group was, on average, younger than the MeshH group. Robotic surgery was performed more often in the MeshH group, possibly reflecting a preference bias by the surgeon towards the robotic route for MeshH, which is technically more challenging than SutureH. We had no intraoperative complications and no blood transfusions in either group.

A variety of meshes were used for the MeshH during the study period. In 97% of MeshH cases, a type 1 macroporous monofilament polypropylene mesh was used. In 4 cases (3%), a macroporous composite mesh with a coating of absorbable material on either side of the mesh was used (Physiomesh; Ethicon, USA). This mesh was later recalled owing to “non-stickiness” and high failure rates.

Several techniques have been described for SutureH and MeshH. Our SutureH involves bilateral shortening of the collagenous intermediate portion of the uterosacral ligaments towards the cervix and may achieve a more anatomical alignment of the uterus, minimising the narrowing of the anterior rectal space. This theoretically corrects elongation of the uterosacral ligaments observed in prolapse while maintaining a normal uterine axis [4]. This is different from the uterosacral ligament “plications” performed at the time of hysterectomy, which is midline approximation and closure of the peritoneum (McCall culdoplasty) or for vaginal vault prolapse, where they are approximated in the midline without plication. The surgeon in this study developed two previous SutureH techniques [11, 12] and our current technique described in this study is a further modification. Based on the objective outcome, our study reports a 95.8% success rate, which is much better than the pooled objective outcome of 70.5% quoted by Nair et al. [15]. This may be largely due to the inclusion of the study by Bedford et al., which had a significantly lower success of 41% than others, which varied between 79 and 100% [18]. The main reason for high failure in Bedford’s study was cervical elongation and uterine bulkiness. Maher et al. reporting a 79% success rate used “no prolapse” as the objective outcome compared with POP-Q stage 2 prolapse used in other studies [11]. There were no ureteric injuries in our study, which compares favourably with vaginal approaches, where visualisation of the ureter is difficult [19]. The peritoneal relaxing incisions may also reduce ureteric “kinking” from tying of the uterosacral sutures.

Our MeshH technique differs from other techniques. We employ a “U-shaped” mesh acting like a “sling” around the isthmus of the uterus. This mitigates the risk of mesh detachment from the uterus. We also attach two mesh straps to the sacral promontory with sutures. Although similar to the “Oxford technique”, the theoretical advantages of our technique are, first, minimising the bulk of the mesh over the anterior cervix, which is in close proximity to the bladder, and second, more robust support with two straps instead of one [20]. Techniques where the points of mesh fixation are the posterior cervix and sacral promontory may result in ante-flection of the uterus. As a result, the posterior cervical mesh attachment may make the anterior cervix and upper anterior vagina more vulnerable to recurrent prolapse and more prone to mesh detachment from the posterior cervix or uterine isthmus [21].

With regard to total vaginal length, both groups were able to maintain the baseline vaginal length. This is a potential advantage for sexual function over vaginal hysterectomy, which generally results in a shorter vaginal length from baseline [22].

There was no difference with respect to subjects’ reported symptoms of prolapse at 1 year. We used subjects’ observation of symptoms over symptom-specific quality of life measurements. However, an overall subjective assessment was undertaken by telephone using PGI-I by two independent investigators not involved in clinical care and blinded to the type of surgery. When contacted, subjects were informed that their participation was voluntary and would not affect clinical care. All the subjects had already been discharged from clinical care at the 1-year review. These factors minimised the risk of participation bias. Even though 34% of the subjects could not be contacted, the distribution was even in the two groups. If those who could not be contacted were all considered as treatment failures the “composite success” rate would be 60.83% (*n*=120) in the MeshH and 56.33% (*n*=40) in the SutureH group (RR=1.08, 95% CI=0.84–1.39, *p*=0.541, NS).

There were no mesh exposures in the MeshH group, similar to the largest reported cross-sectional study for mesh hysteropexy [20]. Pelvic pain was reported by 3% in the MeshH group with no requests for mesh removal. When comparing re-operation rates, more women had surgery than were classed as objective failure with respect to POP-Q point C. Most re-operations for uterine prolapse had POP-Q point C above the hymen. This is consistent with ultrasonography studies that reported a threshold of POP-Q point C of −5 cm correlates with symptoms of prolapse [23]. Although a larger number in the SutureH group had Burch colposuspension, this had no adverse effect on the posterior compartment at 1 year.

The limitations of our study include the non-randomised retrospective methodology, lack of sample size estimation, single surgeon, lack of blinding (except for the investigators at the assessment beyond 1 year), not using validated QoL measures and 34% uncontactable for telephone follow-up. Our strengths are in the comparatively large sample size and being the first direct comparison study of SutureH and MeshH. The confounding bias due to variation in surgical technique is minimised with a single experienced surgeon and surgery not being undertaken during the learning curve. The pragmatic nature of the study, which incorporates women’s and surgeon’s preferences around surgery, provides validity and generalisability in the context of current changing trends in prolapse surgery. Our study may also help in informing the design of future studies measuring expected differences between similar techniques. We also recommend using a single outcome measure rather than a composite outcome as the primary endpoint.

## Conclusions

Laparoscopic and robot-assisted suture hysteropexy and mesh sacral hysteropexy provide women with minimally invasive effective and safe options for uterine preservation during pelvic organ prolapse surgery. When controlling for age, MeshH provides better uterine support than SutureH. However, SutureH gives women a mesh-free option.
